# Evaluation of Dialysis Water Quality at Hospitals in Baghdad, Iraq

**DOI:** 10.5696/2156-9614-10.28.201211

**Published:** 2020-12-07

**Authors:** Yasamen Raad Humudat, Saadi Kadhim Al-Naseri

**Affiliations:** Environment and Water Directorate, Ministry of Science and Technology, Baghdad, Iraq

**Keywords:** dialysis fluid, hemodialysis, water quality

## Abstract

**Background.:**

Dialysis water quality is one of the most important factors for ensuring the safe and effective delivery of hemodialysis. It has been known for over a decade that there might be hazardous contaminants in the water and concentrates used to prepare dialysis fluid. Rigorous international standards for the purification of dialysis fluids have been established, which were used in the present study to compare the quality of dialysis water as there is no national standard for dialysis water quality in Iraq.

**Objectives.:**

There are more than 20 dialysis centers in Iraq, most of which contain similar units for the processing of dialysis water. The present study evaluated the quality of water used for dialysis in four dialysis centers located within Baghdad hospitals.

**Methods.:**

Physical and chemical tests were carried out in the laboratory after sampling water from each dialysis center. Water samples were collected from three locations in each dialysis center. Hospital municipal water samples were collected from the tanks feeding dialysis units; samples of dialysis water were collected from the dialysis water treatment unit outlets; and samples of dialysis water were collected from the distribution network in dialysis rooms.

**Results.:**

The results showed a fluctuation in the quality of the dialysis water (dialysis water and water from the dialysis distribution network), indicating that it is unacceptable compared to international standards. Chemical analysis showed that 75% of the dialysis water had elevated aluminum concentrations. Chemical analysis also found that dialysis water had elevated concentrations of free residual chlorine at some dialysis centers.

**Conclusions.:**

All hemodialysis centers need careful monitoring and preventive maintenance to ensure good water quality. In addition, it is important to revise the design of the water treatment units according to water quality.

**Competing Interests.:**

The authors declare no competing financial interests

## Introduction

Water treatment plays a crucial role in maintaining human health and welfare.^1^ Municipal water (tap) is the main source of water used in hemodialysis centers. This water passes through several levels of pretreatment to get rid of any contaminants.^2^ Water contamination can lead to anemia, changes in blood pressure and acid-base balance, neurological problems, bone disease, and more, and patients may experience acute or chronic problems under standard dialysate.^3^

In 2018, the Iraqi Ministry of Health reported that there were 19 dialysis centers distributed in all provinces except the Kurdistan region. In Baghdad alone, there are 11 dialysis centers with about 320±61 regularly registered patients. Each center has an average of 37±8 hemodialysis machines. The total production capacity for each center varies between 1.5–4.5 m^3^/d, depending on the daily operating hours, ranging from 18 to 24 hours. Units to treat municipal water in those centers work in the same manner, using reverse osmosis (RO). The output of this treatment is dialysis water, which is then mixed with dialysate for dialysis treatment.

Patient hemodialysis sessions usually take place 3 times a week for approximately 3–4 hours a session.^4^ Consumption is approximately 90–192 L of dialysis water per patient per session depending on the flow rate of the dialysate and the percentage of water rejected during the RO process.^3^ Dialysis fluid volume rises to 580–860 L per week for hemodialysis patients. Ensuring dialysis fluid quality is an important aspect of this type of treatment considering the frequent large quantities used for each patient.^5^

In particular, contamination with chemical, bacterial, and associated endotoxins may threaten the health of a patient under dialysis, thus the International Organization for Standardization (ISO) and a similar standard from both the American National Standards Institute (ANSI) and the Association for the Advancement of Medical Instrumentation (AAMI) have issued standards and recommendations to ensure water quality needed to reduce chemical hazards for hemodialysis patients.^6,7^ These standards were used to compare the study results to determine the quality of the dialysis water and to suggest the best strategy to ensure that this water complies with the international standard. The international standard was considered as no national dialysis water standards have yet been issued in Iraq. The present study evaluated the quality of water used for dialysis in four dialysis centers located within Baghdad.

## Methods

The sampling process involved the collection of water samples from each of the four hemodialysis centers (Al-Kindi, Baghdad Teaching, Al-Imamain Al-Kadhimain, and Al-Yarmouk) during the four seasons (spring, summer, autumn and winter) from March 2018 to February 2019. Samples were collected in a clean 500 mL glass bottle and used for chemical tests. Samples were transported within 1–2 hours to the laboratories of the Ministry of Science and Technology in Baghdad for chemical tests. Another 25 mL was collected in a clean flask for the on-site examination of several variables (temperature, pH, electrical conductivity (EC), total dissolved solids (TDS), total hardness (TH), turbidity, and free chlorine).

### Sampling locations

Water samples were collected from three location types in each dialysis center: 1) municipal water supplied to the dialysis center by each hospital was collected from the tank feeding the unit, 2) dialysis water, and 3) water from the distribution network in dialysis rooms. The distribution network carries the dialysis water to the patient's dialysis machines. Sixteen samples were collected from each type (4 samples a year from 4 hospitals), following the sampling procedure suggested by the Northwest Renal Network.^8^

**Abbreviations**
*AAMI*: Association for the Advancement of Medical Instrumentation
*ANSI*: American National Standard Institute
*EC*: Electrical conductivity
*ISO*: International Organization for Standardization
*RO*: Reverse osmosis
*TDS*: Total dissolved solids
*TH*: Total hardness

### Chemical analysis

All field and laboratory measurements were conducted using portable instruments manufactured by the Hach Company (Loveland, CO, USA). pH was measured using a portable pH/ISE meter model SensIon1 with a platinum series pH electrode, EC was measured using SensIon5, and free chlorine was measured using the N,N-diethyl-p- phenylene-diamine colorimetric method with DR890 colorimeter. Samples were then transported to the laboratory for thorough chemical analysis, including (calcium (Ca) and magnesium (Mg)). The measurements were performing using an atomic absorption spectrometer.

### Statistical analysis

Analysis of variance test (ANOVA) was used to evaluate the significant effects of the different parameters at a significant *P*-value level of 0.05. Statistical analysis was carried out using Microsoft Excel 2010.

## Results

Four dialysis centers were monitored for four seasons and water samples were collected for physical and chemical analysis. Municipal water results are presented in Table 1 with a comparison with Iraqi drinking water standards.^9^ Measured values for dialysis water in treatment units are shown in Tables 2 and 3 with a comparison with International Organization for Standardization/Association for the Advancement of Medical Instrumentation (ISO/ANSI/AAMI)^6,7^ standard values for produced dialysis water. The analyses included most of the AAMI standard chemical substances plus several others, including pH, temperature, EC, turbidity, TDS, TH, free residual chlorine, Ca, Mg, and aluminum (Al).

**Table 1:** Seasonal Variation in Physiochemical Variables of Municipal Water Across Hospital Dialysis Centers During the Study Period Compared to Iraqi Drinking Water Standards 9

**Variable**	**Hospitals**	**Spring**	**Summer**	**Autumn**	**Winter**	**Significant differences**
Temperature	Al-Kindi	26	23	29	18	0.02*
	Baghdad Teaching	22	23	25	21	
	Al-Imamain Al-Kadhimain	23	24	30	18	
	Al-Yarmouk	23	26	20	20	
PH 6.5–8.5^9^	Al-Kindi	6.8	7.46	7.65	7.02	NS
	Baghdad Teaching	7.5	7.7	7.22	6.9	
	Al-Imamain Al-Kadhimain	7.23	6.5	6.64	7.44	
	Al-Yarmouk	7.4	7.23	8.08	7.17	
EC μS/cm	Al-Kindi	876	1186	899	979	NS
	Baghdad Teaching	762	939	898	832	
	Al-Imamain Al- Kadhimain	725	768	547	732	
	Al-Yarmouk	1021	1008	507	926	
TDS 1000 mg/l^9^	Al-Kindi	486	676	410	584	2.43*
	Baghdad Teaching	432	529	410	504	
	Al-Imamain Al-Kadhimain	416	409	298	438	
	Al-Yarmouk	588	561	254	545	
Turbidity 5 mg/l^9^	Al-Kindi	7.09	1.34	8.25	10.5	NS
	Baghdad Teaching	1.83	4.9	6.11	8.78	
	Al-Imamain Al-Kadhimain	1.57	4.38	1.64	1.73	
	Al-Yarmouk	2.54	2.61	4.11	1.89	
TH 500 mg/l^9^	Al-Kindi	440	500	450	500	NS
	Baghdad Teaching	200	160	380	260	
	Al-Imamain Al-Kadhimain	300	500	200	173	
	Al-Yarmouk	500	460	254	480	
Free chlorine 0.2 mg/l^9^	Al-Kindi	0.25	0.01	0.05	0.05	NS
	Baghdad Teaching	0.2	0.01	0.15	0.15	
	Al-Imamain Al-Kadhimain	0.17	0.2	0	0.43	
	Al-Yarmouk	0.01	0.07	0.05	0.93	
Ca 150 mg/l^9^	Al-Kindi	48	111	169	66.87	
	Baghdad Teaching	0.2	1.51	30	33.43	NS
	Al-Imamain Al-Kadhimain	90	1.51	58	40	
	Al-Yarmouk	120	50	15	46	
Mg 100 mg/l^9^	Al-Kindi	40	96	36	24.24	
	Baghdad Teaching	24	85	33	23	
	Al-Imamain Al-Kadhimain	20	85	29	220	NS
	Al-Yarmouk	49	45	26	179	
A1 0.2 mg/l^9^	Al-Kindi	UDL	UDL	0.015	0.0047	NS
	Baghdad Teaching	UDL	UDL	0.02	0.04	
	Al-Imamain Al-Kadhimain	UDL	UDL	0.05	0.03	
	Al-Yarmouk	UDL	UDL	0.01	0.01	

^*^ = (*P* < 0.05) significant ^#^=Iraq standard

Abbreviations: NS, not significant; UDL, under detection limit.

**Table 2:** Seasonal Variation of Physiochemical Variables in Dialysis Water Across Hospital Dialysis Centers Compared to International Standards During the Study Period ^6,7^

**Variables**	**Hospitals**	**Spring**	**Summer**	**Autumn**	**Winter**	**Significant differences**
Temperature °C	Al-Kindi	22	24	29	18	0.01*
	Baghdad Teaching	21	24	28	20	
	Al-Imamain Al-Kadhimain	23	23	27	20	
	Al-Yarmouk	23	27	33	21	
pH	Al-Kindi	5.4	7.56	7.7	6.59	NS
	Baghdad Teaching	5.97	6.9	8.4	7.35	
	Al-Imamain Al-Kadhimain	5.9	5.7	7.06	7	
	Al-Yarmouk	7.84	7.57	8.73	7.5	
EC μs/cm	Al-Kindi	21	22	60	14	NS
	Baghdad Teaching	7	27	22	14	
	AL-Imamain Al-Kadamain	20	24	19	20	
	Al-Yarmouk	92.1	199	115	16	
Turbidity NTU	Al-Kindi	1.57	1.43	1.19	1.72	NS
	Baghdad Teaching	0.73	1.66	1.81	2.08	
	Al-Imamain Al-Kadhimain	1.23	1.43	0.7	1.45	
	Al-Yarmouk	1.34	0.91	0.36	1.01	
TDS mg/l	Al-Kindi	12	13	28	8	NS
	Baghdad Teaching	4	15	10	8	
	Al-Imamain Al-Kadamain	11	13	9	12	
	Al-Yarmouk	53.2	108	58	10	
TH mg/l	Al-Kindi	80	100	190	8	NS
	Baghdad Teaching	25	20	20	18	
	Al-Imamain Al-Kadhimain	12	100	220	15	
	Al-Yarmouk	18	240	58	18	
Free chlorine 0.1 mg/l^6,7^	Al-Kindi	0.04	0.02	0.02	0.07	
	Baghdad Teaching	0.02	0	0.02	0.07	NS
	Al-Imamain Al-Kadhimain	0	0.17	0	0.12	
	Al-Yarmouk	0	0.03	0.04	0.53	
Ca 2 mg/l^6,7^	Al-Kindi	0.1	UDL	1.01	0.26	NS
	Baghdad Teaching	0.5	UDL	UDL	1.1	
	Al-Imamain Al-Kadhimain	3	UDL	UDL	4	
	Al-Yarmouk	5	3	0.32	0.1	
Mg 4 mg/l^6,7^	Al-Kindi	4	0.11	0.22	0.06	NS
	Baghdad Teaching	0.25	0.1	0.05	0.42	
	Al-Imamain Al-Kadhimain	1	0.09	0.08	3.26	
	Al-Yarmouk	1.4	15	0.2	0.26	
Al 0.01 mg/l^6,7^	AL-Kindi	0.03	0.015	0.03	0.032	NS
	Baghdad Teaching	0.045	0.005	0.04	0.035	
	Al-Imamain Al-Kadamain	0.015	0.005	0.04	0.03	
	Al-Yarmouk	UDL	0.015	0.01	0.02	

* = (*P* < 0.05) significant ^#^ = International Organization for Standardization/Association for the Advancement of Medical Instrumentation/American National Standard Institute standard

Abbreviations: NS, not significant; UDL, under detection limit; NTU, nephelometric turbidity units.

**Table 3:** Seasonal Variation of Physiochemical Variables in Water Samples from Dialysis Distribution Network Hospitals Compared to International Standards During the Study Period ^6,7^

**Variables**	**Hospitals**	**Spring**	**Summer**	**Autumn**	**Winter**	**Significant differences**
Temperature °C	Al-Kindi	24	24	26	18	0.01*
	Baghdad Teaching	22	24	28	20	
	Al-Imamain Al-Kadhimain	22	23	20	20	
	Al-Yarmouk	22	26	32	21	
pH	Al-Kindi	5.6	7.26	7.2	6.66	3.53*
	Baghdad Teaching	5.28	7.66	8.25	7.5	
	Al-Imamain Al-Kadhimain	5.4	6.7	7.02	7.02	
	Al-Yarmouk	7.63	7.41	9.42	7.14	
EC μS/cm	Al-Kindi	23	23	31	18	NS
	Baghdad Teaching	14	76	67	12	
	Al-Imamain Al-Kadhimain	15	22	17	17	
	Al-Yarmouk	140	210	67	15	
Turbidity NTU	Al-Kindi	2.3	1.54	1.14	1.45	NS
	Baghdad Teaching	0.93	2.85	1.89	1.72	
	Al-Imamain Al-Kadhimain	1.33	1.42	1.2	1.86	
	Al-Yarmouk	2	1.12	2.12	1.38	
TDS mg/l	Al-Kindi	13	11	15	11	NS
	Baghdad Teaching	8	54.8	30	7	
	Al-Imamain Al-Kadhimain	9	24	9	10	
	Al-Yarmouk	87	115	34	9	
TH mg/1	Al-Kindi	120	80	200	20	NS
	Baghdad Teaching	35	20	50	9	
	Al-Imamain Al-Kadhimain	20	120	100	19	
	Al-Yarmouk	18	280	34	14	
Free chlorine 0.1 mg/l^6,7^	Al-Kindi	0.01	0	0.01	0.07	NS
	Baghdad Teaching	0.02	0	0.02	0.07	
	Al-Imamain Al-Kadhimain	0	0	0	0.12	
	Al-Yarmouk	0	0.08	0.02	0.53	
Ca 2 mg/l^6,7^	Al-Kindi	0.3	0.1	0.24	1.003	NS
	Baghdad Teaching	1	UDL	UDL	0.34	
	Al-Imamain Al-Kadhimain	6	UDL	UDL	0.1	
	Al-Yarmouk	8	3.2	0.32	0.1	
Mg 4 mg/l^6,7^	Al-Kindi	0.2	0.1	0.18	0.135	NS
	Baghdad Teaching	0.56	0.1	UDL	0.099	
	Al-Imamain Al-Kadhimain	1.2	0.1	0.06	0.41	
	Al-Yarmouk	1.6	17	0.1	0.05	
Al 0.01 mg/l^6,7^	Al-Kindi	0.05	0.071	0.004	0.004	NS
	Baghdad Teaching	0.03	0.004	0.004	0.033	
	Al-Imamain Al-Kadhimain	0.045	0.004	0.05	0.03	
	Al-Yarmouk	UDL	0.013	0.03	0.02	

* =(P< 0.05) significant *#*= International Organization for Standardization/Association for the Advancement of Medical Instrumentation/American National Standard Institute standard

Abbreviations: NS, not significant; UDL, under detection limit; NTU, nephelometric turbidity units.

The results of the chemical analyses of municipal water are presented in Table 1. Statistical analysis showed no seasonal significant differences among the different dialysis centers at (*P* < 0.05) for water variables except for temperature and TDS. Electrical conductivity, pH, turbidity, TH, free residual chlorine, Ca, Mg, and Al in water samples were compared to the recommended concentrations in Iraqi drinking water standards. Furthermore, the lowest value of free chlorine (0 mg/l) was measured in the summer at Al-Imamain Al-Kadhimain hospital, while the highest level was measured in the winter at Al-Yarmouk hospital and 0.9 mg/l. The Iraqi standard recommends minimum free chlorine be no lower than 0.3 mg/l.^9^

In addition, turbidity, Ca, and Mg concentrations exceeded the maximum level suggested by Iraqi drinking water standards in some centers by about 19% for both turbidity and Ca, and 13% for Mg. However, these differences were non-significant, while differences in water temperature and TDS were significant across the centers.

According to Table 2, the statistical analysis showed no seasonal significant differences for dialysis water at (*P* > 0.05) across the different dialysis centers in the hospitals for all variables except water temperature. Furthermore, free residual chlorine and Al concentrations exceeded the maximum level suggested by the (ANSI/AAMI/ISO) standards by 13% and 75% in some centers, respectively.

Finally, as seen in Table 3, there were no seasonal significant differences for water samples from the dialysis distribution network at (*P* > 0.05) across the different dialysis centers except for water temperature and pH at the hospitals during the study period. However, free residual chlorine and Al concentrations exceeded the maximum level suggested by ISO/AAMI by 13% and 63% in some of the centers, respectively.^6,7^

## Discussion

The chemical analysis showed that municipal water quality did not comply with Iraqi standards and criteria guidelines for several substances, including turbidity, free residual chlorine, Ca and Mg *(Table 1).* Comparison of the chemical analysis results with the quality of dialysis water and water samples from the distributed dialysis network characteristics in Tables 2 and 3 indicates that the units have good rejection rates, indicating good performance of RO membranes (except for Al-Yarmouk hospital, due to age). Nevertheless, poor maintenance and seasonal variation of municipal water quality make one-stage RO unsuitable for removing all chemical pollutants. Al-Yarmouk hospital showed a high deviation of free residual chlorine concentration from the standard value (0.1 mg/l).^6,7^ The same unit also had the highest TH and TDS values for water, along with elevated EC values.

There were significant differences in hospital municipal water temperatures across the seasons, with an inverse effect on the efficiency of the water treatment units and in turn affecting the TDS results. This is mainly due to seasonal differences, and differences in pipes, storage tanks (tanks type) for municipal water distribution systems, and location of tanks in hospitals.^10^ In addition, municipal water temperature affects the integrity of RO and particle filters, which are set by the manufacturer to a maximum operating temperature, usually less than 35°C.^11^

In addition, EC value can be a useful indicator of the performance of the water treatment unit, because water with an EC value lower than 25 μS/cm means a low TDS value for the same water.^12^ It should be noted that patient vomiting and nausea have been reported to be more frequent when EC values in dialysis water are increased.^10^

The present study confirmed that municipal water in Baghdad dialysis centers meets the Iraqi standard for TDS of 1000 mg/l.^9^ No comparison was done with World Health Organization (WHO) guidelines as there is no guideline value for TDS because it is “not of health concern at levels found in drinking water”.^13^

In general, all the results showed a reduction in TH levels after dialysis water treatment. Reverse osmosis is able to decrease hardness, although high levels can adversely affect the RO membrane and decrease its life as it is quickly fouled by hard water (water with a high mineral content).^14^ Therefore, pretreatment must be used to protect RO membranes in dialysis units.

Free chlorine was measured for the feed and produced dialysis water. Elevated concentrations of free chlorine (higher than 0.1 mg/l) in the produced dialysis water at Al-Yarmouk hospital were recorded due to the addition of hypochlorite, which is widely used in dialysis centers to disinfect water networks. The lack of free chlorine in the municipal water to the dialysis water treatment units indicated that an insufficient amount of chlorine was initially added to the water. The reason for the different levels in municipal water was possibly due to municipal water treatment plant disinfection practices and climatic conditions during the study period.^15^

The deviation from other contaminants (Ca and Mg) was not significant. It is important to monitor water hardness after softening in the treatment unit and to use quality sodium chloride for regeneration as inefficient softening will lead to an increase in the concentration of Ca and Mg in the RO unit. Accordingly, failing to comply with international standards can be attributed to the aging of the RO membrane. Nevertheless, softeners should remove Ca^+2^ and Mg^+2^ ions before the RO membrane, but the results of the present study indicate that this treatment was not performing well across all seasons.^10^

In addition, elevated Al is expected as the current practice of clarifying municipal water in Iraq is to use aluminum sulfate as a coagulant. This can lead to feeding dialysis centers with municipal water contaminated with a high concentration of Al (greater than 0.2 mg/l).^9^ A number of studies have shown that Al toxicity is dangerous and can cause severe morbidity and even mortality.^16^ In some parts of the world, outbreaks of unexplained, progressive dementia, often leading to death, have occurred because of substantial Al contamination of local water supplies.^16^

A single-stage RO unit was used by all four dialysis centers. It is clear from the chemical analysis that one-stage RO is insufficient, and it is important to adjust the design of water treatment systems to include a second-stage RO unit after the first. The drain of this stage can be returned to the first stage water tank and therefore no water losses will occur. This will reduce the production capacity of the units but will make them safer and prevent an increase in the concentration of chemical pollutants.

## Conclusions

Field monitoring study results showed that municipal and dialysis water did not meet the standard requirements for the international dialysis fluid specifications. Further studies are needed to investigate other types of potential contamination (chemical and microbial) to assess the overall quality of dialysis water treatment units in Baghdad.

## References

[1] Timmers L, Thong M, Dekker FW, Boeschoten EW, Heijmans M, Rijken M, Weinman J, Kaptein A (2008). Illness perceptions in dialysis patients and their association with quality of life. Psychol Health [Internet].

[2] Montanari LB, Sartori FG, Cardoso MJ, Varo SD, Pires RH, Leite CQ, Prince K, Martins CH (2009). Microbiological contamination of a hemodialysis center water distribution system. Rev Inst Med Trop Sao Paulo [Internet].

[3] Amato RL (2005). Water treatment for hemodialysis--updated to include the latest AAMI standards for dialysate (RD52: 2004) continuing. Nephrol Nurs J.

[4] Tentori F, Zhang J, Li Y, Karaboyas A, Kerr P, Saran R, Bommer J, Port F, Akiba T, Pisoni R, Robinson B (2012). Longer dialysis session length is associated with better intermediate outcomes and survival among patients on in-center three times per week hemodialysis: results from the Dialysis Outcomes and Practice Patterns Study (DOPPS). Nephrol Dial Transplant [Internet].

[5] Coulliette AD, Arduino MJ (2013). Hemodialysis and water quality. Semin Dial [Internet].

[6] (2014). Water treatment equipment for hemodialysis and related therapies.

[7] (2014). ISO 267222014 Water treatment equipment for haemodialysis applications and related therapies [Internet].

[8] (2005). Monitoring your dialysis water treatment system [Internet]. https://www.nwrn.org/files/WaterManual.pdf.

[9] (2009). Iraqi Standard of Drinking Water No. 417.

[10] Hamoudat YR (2020). Evaluating dialysis fluids properties and its effects on hemodialysis patients at several hospitals in Baghdad [dissertation].

[11] Dheda S, Eps C, Hawley C, Johnson DW, Suzuki H (2015). Water treatment for centre and home-based haemodialysis. Updates in hemodialysis [Internet].

[12] (2018). Water for dialysis: a guide for in-center, satellite and home haemodialysis in NSW [Internet]. https://www.aci.health.nsw.gov.au/__data/assets/pdf_file/0007/306088/water-for-dialysis2018.pdf.

[13] (2017). Guidelines for drinkingwater quality [Internet].

[14] Benham B, Ling E (2019). Virginia household water quality program household water treatment [Internet].

[15] Besic A, Obradovic Z, Dautbegovic A, Obradovic A (2017). The effect of temperature and chlorine residual on the presence of Legionella spp. in water systems of public and tourist facilities. J Health Sci [Internet].

[16] Al-Naseri SK, Mahdi ZM, Hashim MF (2013). Quality of water in hemodialysis centers in Baghdad, Iraq. Hemodial Int [Internet].

